# The Impact of Hemolytic Processes Due to Extracorporeal Support Therapy on the Serum Concentration of the Neuronal Marker Protein NSE

**DOI:** 10.3390/ijms27041910

**Published:** 2026-02-17

**Authors:** Daniel Ebert, Kurt Henrik Janke, Julia Schumann

**Affiliations:** University Clinic and Outpatient Clinic for Anesthesiology and Operative Intensive Care, University Medicine Halle (Saale), Franzosenweg 1a, 06112 Halle (Saale), Germany

**Keywords:** NSE, ECMO, hemolysis, clinical marker, brain damage

## Abstract

Neuron-specific enolase (NSE) is a marker used to assess neurological impairment. Notwithstanding, the release of NSE into the circulation can also originate from erythrocytes and thrombocytes, signifying that even mild instances of hemolysis have the potential to induce heightened serum NSE levels. The present study addresses the question of whether the serum NSE level is a reliable parameter for assessing potential brain damage in patients undergoing extracorporeal membrane oxygenation (ECMO). To this end, NSE values of all non-resuscitated ECMO patients treated at our clinic from January 2020 to March 2022 were retrospectively evaluated. Serum NSE levels were found to be median 35.95 µg/L, with significant intrapersonal variability during ECMO therapy. A comparative analysis in ECMO patients with and without diagnosed brain damage revealed no statistically significant differences. In contrast, the concurrent measurement of serum LDH and NSE levels exhibited a significant positive correlation (Spearman Rho 0.69), indicating that the elevated serum NSE levels exhibited by patients devoid of cerebral impairment were attributable to the occurrence of ECMO-induced hemolysis. Consequently, the prognostic value of serum NSE levels in patients undergoing ECMO is restricted. The data also demonstrate that individual measurements of serum NSE levels in ECMO patients should be regarded as snapshots with only limited significance.

## 1. Introduction

Neuron-specific enolase, otherwise known as NSE, is an enzyme involved in the process of glucose metabolism [1,2]. It is primarily expressed in the nervous tissue, particularly by neurons, and serves as a biomarker for neurological damage that can be measured quickly and reproducibly in the blood [3,4]. In clinical practice, the determination of NSE is therefore part of a comprehensive approach for evaluating the neurological prognosis of resuscitated patients following cardiac arrest, as well as those undergoing heart and thoracic surgery [1,5,6,7]. Elevated NSE levels are described as correlating with the occurrence of serious neurological complications and higher in-hospital mortality, especially during treatment with veno-arterial (VA)-ECMO [2,4,6,8,9]. However, this correlation must be carefully interpreted in the context of no-flow time-induced hypoxemia after cardiac arrest. Although the optimal NSE threshold under VA-ECMO remains unclear, recent ERC guidelines suggest using NSE levels greater than 60 µg/L after 48 and/or 72 h to predict poor neurological outcomes [7]. It is not known whether the specified NSE cut-off value is suitable for patients with veno-venous (VV)-ECMO. The Extracorporeal Life Support Organization (ELSO) guideline for ECMO management [10] does not contain any recommendations regarding NSE.

NSE is not exclusively present in neurons; it is also found in erythrocytes and thrombocytes [1,3,4,9,11,12]. Laboratory studies have demonstrated that even slight instances of hemolysis can result in an elevation of NSE levels [13,14,15]. This aspect is pertinent to the clinical interpretation of measured NSE values. Research has demonstrated that the implementation of mechanical long-term circulatory support systems (i.e., ventricular assist devices, VAD) results in an elevation in NSE levels, which is concomitant with the concurrent increase in laboratory parameters of hemolysis [3]. However, in the context of short-term circulatory support for critically ill patients using extracorporeal membrane oxygenation (ECMO) and/or Impella therapy, this relationship has not been sufficiently investigated. Consequently, the current state of research precludes a definitive conclusion regarding the prognostic value of NSE in predicting neurological outcomes in ECMO patients. Elevated NSE levels, which have been linked to suboptimal neurological outcomes in resuscitated patients following cardiac arrest [7], may primarily be due to device-associated hemolysis rather than direct brain damage. This aspect assumes particular significance in acute situations with highly elevated NSE levels, i.e., following the implantation of extracorporeal devices and the necessity of profound sedation, as it is inherently challenging to adequately assess the patient’s neurological status in such circumstances. The worst-case scenario posits that the assumption of severe brain injury could potentially result in an unjustified modification of treatment objectives and premature discontinuation of life-sustaining measures by intensive care physicians. The present study therefore investigates blood concentrations of NSE in patients receiving ECMO therapy. In essence, the objective of this study is to assess the utility of NSE as a marker of brain injury in patients undergoing temporary extracorporeal life support.

## 2. Results

### 2.1. Characteristics of the Study Population

**Figure 1:**
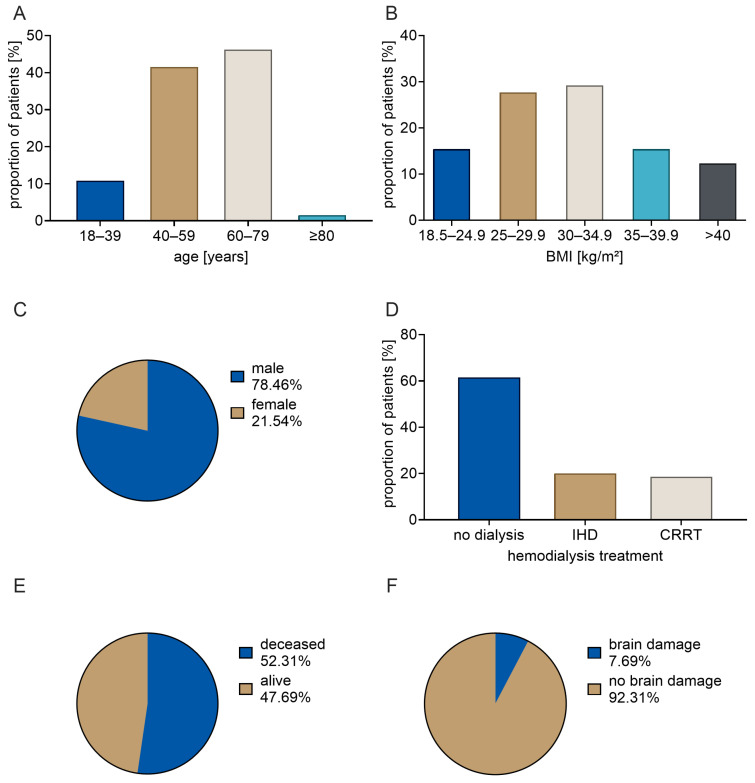
Characteristics of the study cohort (*n* = 65). (**A**): Age distribution. (**B**): BMI distribution. (**C**) Gender distribution. (**D**): Frequency of hemodialysis requirement. (**E**): Frequency of patient mortality. (**F**): Frequency of brain damage.

The present study was based on a comprehensive analysis of all ECMO patients with documented NSE values who were treated in the intensive care unit (ICU) of the University Clinic for Anesthesiology and Operative Intensive Care Medicine at University Medicine Halle (UMH) during the defined study period (January 2020 to March 2022) (*n* = 65; see Figure 1). The majority of patients were male. Furthermore, an elevated proportion of the patients observed were within the age group of 40–79 years. A total of 15.4% of patients were found to be of normal weight. In 84.6% of cases, an elevated body mass index (BMI) above 24.9 was diagnosed, while no cases of underweight (BMI < 18.5) were found. The median BMI recorded was 31.0. Concomitant dialysis was mandated in 38.5% of cases, with 13 patients receiving intermittent hemodialysis (IHD) and 12 patients receiving continuous renal replacement therapy (CRRT). At the conclusion of the ECMO therapy, it was observed that 33 patients exhibited severe consciousness impairment, as defined by a score below 9 on the Glasgow Coma Scale (GCS). Furthermore, fifteen patients exhibited mild to moderate consciousness impairment (GCS 9–13 points), while seventeen patients demonstrated no consciousness impairment (GCS 14–15 points). Brain damage, i.e., documented ICD-10 codes I61 or I63, was evident in five cases. Our study cohort does not include other forms of brain damage, such as compression of the brain (G93.5) or anoxic brain damage (G93.1). Notably, a mere 31 out of the 65 patients were discharged alive.

**Figure 2:**
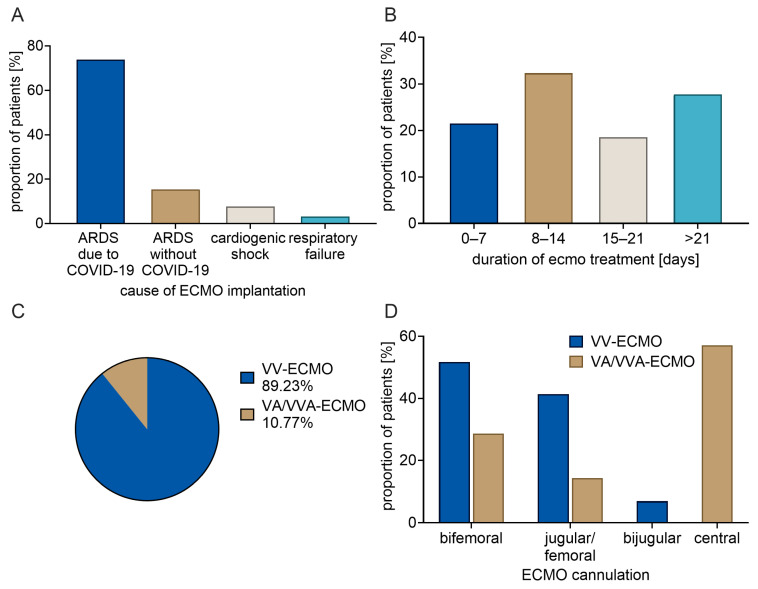
Details on ECMO therapy in the study cohort (*n* = 65). (**A**): Frequency of individual ECMO causes. (**B**): Distribution of ECMO treatment duration. (**C**): Frequency of ECMO types. (**D**): Frequency of cannulation types.

Details on ECMO therapy are given in Figure 2. In the majority of cases, the indication for ECMO therapy was acute respiratory distress syndrome (ARDS). The remaining primary diagnoses included cardiogenic shock (7.7% of cases) and respiratory failure (3.1% of cases). VV-ECMO was conducted in 58 of the 65 patients, with cannulation achieved in 51.7% of cases as bifemoral, in 41.4% as jugular/femoral, and in 6.9% as bijugular. Seven of the 65 patients underwent VA- or veno-veno-arterial (VVA)-ECMO, with cannulation performed via the bifemoral route in 28.6% of cases, the jugular/femoral route in 14.3% of cases, and the central route in 57.1% of cases. The documented duration of ventilation therapy ranged from up to one week in 21.5% of cases, up to two weeks in 32.3% of cases, up to three weeks in 18.5% of cases, and ECMO therapy was maintained for a duration greater than three weeks in 27.7% of cases. In 21 of the 65 cases (32.3%), an oxygenator change was necessary during the course of ECMO therapy.

### 2.2. The NSE Value of the Individual ECMO Patients Exhibits Inconsistency over the Course of Therapy

**Figure 3:**
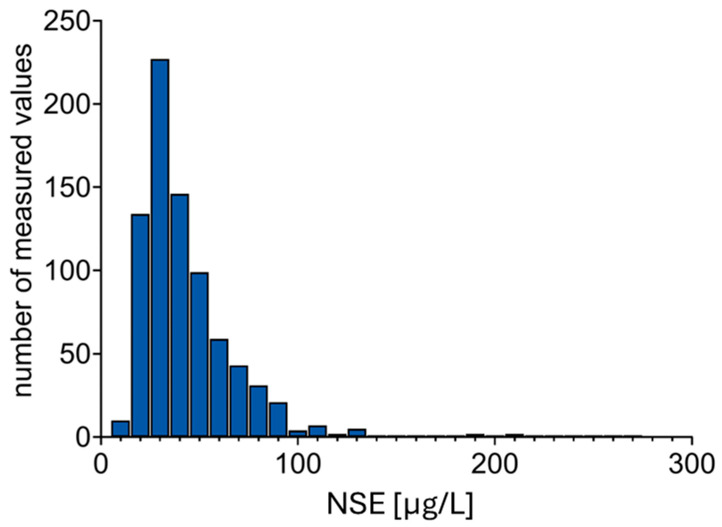
Distribution of measured NSE concentrations within the study cohort.

NSE values were documented in the entire cohort of ECMO patients examined, with a median value of 35.95 µg/L and an interquartile range (IQR) of 18.50. The lowest observed value was 9.4 µg/L, and the highest was 269 µg/L (Figure 3). It is important to acknowledge that, in addition to the anticipated variability between individuals, there were substantial fluctuations in NSE values within individuals over the course of ECMO treatment. On average, the coefficient of variation was calculated to be 37%. These results suggest that individual serum NSE measurements in ECMO patients should be considered snapshots, as their informative value is limited. Notably, the dynamics of the NSE measurements did not display a clear trend. In both patients with and without diagnosed brain damage, repeated measurements of NSE concentration during ECMO therapy showed a spread of values in both directions. In other words, the course of the measurements followed a zigzag pattern. There was no distinguishable difference in the dynamics of the measured values between patients with and without brain damage.

### 2.3. The Prognostic Utility of NSE Is Limited in Patients Undergoing ECMO Therapy

**Figure 4:**
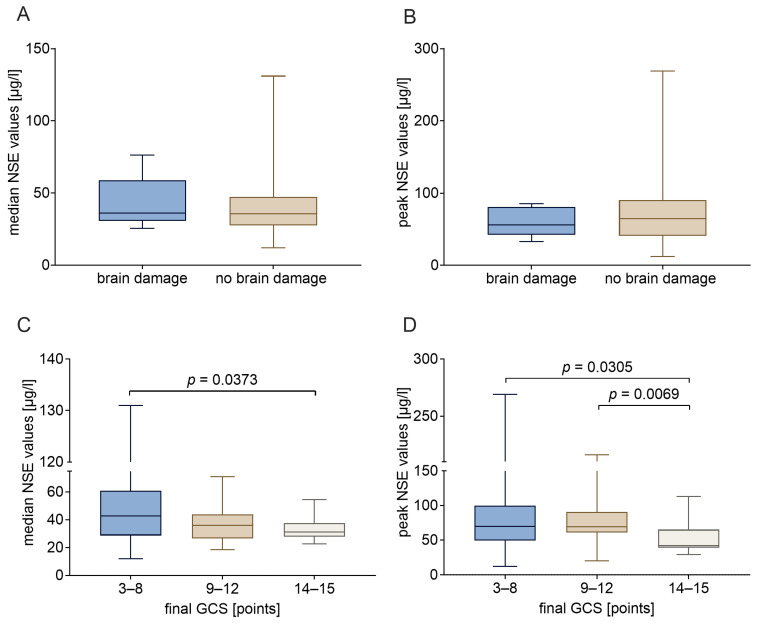
NSE values of ECMO patients stratified according to neurological status. Diagrams (**A**,**B**) compare patients diagnosed with and without brain damage, displaying (**A**) median values and (**B**) maximum values. Diagrams (**C**,**D**) compare patients according to their level of consciousness at the time of discontinuation of ECMO therapy (assessed using the Glasgow Coma Scale, GCS), showing (**C**) median values and (**D**) maximum values. Statistical analysis was performed using GraphPad Prism 10 (GraphPad Software, La Jolla, CA, USA). A *p*-value less than 0.05 was considered to indicate significant differences. Significant disparities were identified through the implementation of a Mann–Whitney test.

The objective of this study was to assess the prognostic relevance of NSE in ECMO patients with respect to the presence of brain damage. To this end, both the median and peak values obtained for each patient were utilized. Figure 4A,B present a comparison of the NSE values of ECMO patients diagnosed with brain damage according to ICD-10 codes I61 or I63 with those of ECMO patients without such a diagnosis. The analysis revealed that neither the median values nor the maximum values exhibited a statistically significant discrepancy between patients with and without an underlying ICD diagnosis. A slightly more differentiated picture emerged when stratifying according to the GCS at the time of discontinuation of ECMO therapy (Figure 4C,D). Patients without clouding of consciousness at the end of ECMO (GCS 14–15) exhibited significantly lower NSE values. However, the substantial variation in the measured values hinders the establishment of a clear separation between the patient groups. Examining the median NSE values revealed the following confidence intervals and effect size when comparing patients with and without severe impairment of consciousness: 31.1 µg/L (95% CI: 28.0–34.5 µg/L) versus 42.7 µg/L (95% CI: 33.7–55.5 µg/L), with an r_rb_-value of 0.36. The comparison of identical patient groups, taking into account the NSE peak values, yielded the following results: 42.1 µg/L (95% CI 39.5–58.9 µg/L) versus 70.0 µg/L (95% CI 57.3–88.3 µg/L); r_rb_ = 0.38.

Additionally, the inability to differentiate between NSE measurements from patients with and without neurological damage for use in clinical practice manifested when stratification was conducted using the cut-off values specified in the ERC guidelines. Within the patient cohort under study, NSE values exceeding 60 µg/L were observed in 36 individuals, while NSE values surpassing even 90 µg/L were detected in 15 individuals. However, only 2 of the 36 individuals (i.e., 5.6%) with NSE values >60 µg/L and none of the 15 individuals with NSE values >90 µg/L were also diagnosed with brain damage. Nonetheless, 14 of the 36 individuals (38.9%) with NSE levels >60 µg/L and 4 of the 15 individuals (26.7%) with NSE levels >90 µg/L were discharged alive from the ICU following the completion of ECMO therapy. Of the 36 individuals with NSE levels >60 µg/L, 4 (11.1%) had no impairment of consciousness (i.e., GCS 14–15) at the time of discharge from the ICU, while of the 15 individuals with NSE levels >90 µg/L, 1 (6.7%) had no impairment of consciousness at the time of discharge from the ICU.

### 2.4. NSE Serum Values Are Influenced by Hemolytic Processes in ECMO Patients

**Figure 5:**
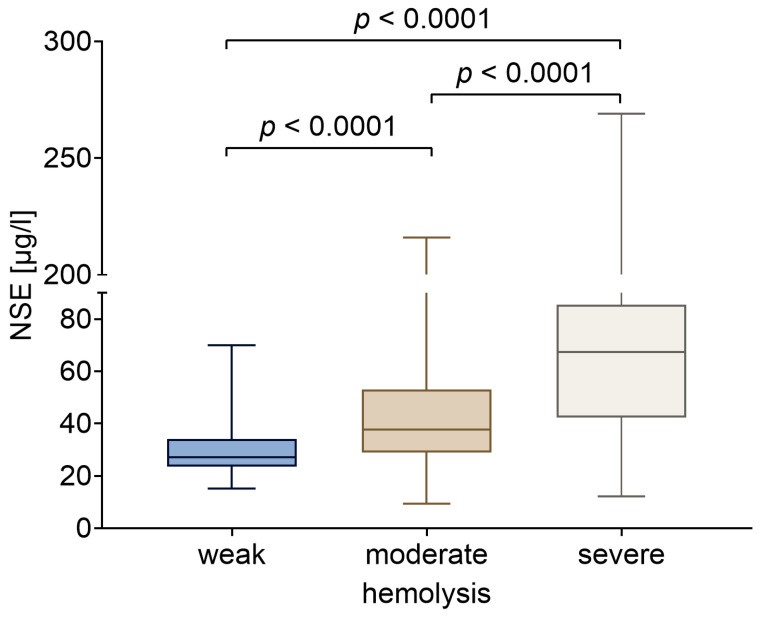
NSE values of ECMO patients stratified according to hemolysis status (mild, moderate, severe). Statistical analysis was performed using GraphPad Prism 10 (GraphPad Software, La Jolla, CA, USA). A *p*-value less than 0.05 was considered to indicate significant differences. Significant disparities were identified through the implementation of a Mann–Whitney test.

In patients devoid of brain damage, the occasionally elevated serum NSE levels imply a source of NSE other than the brain. It was previously established that, in addition to neurons, erythrocytes may serve as a source of NSE release. Therefore, elevated serum levels of NSE might be indicative of hemolytic processes induced by ECMO. Indeed, laboratory testing of standard hemolysis parameters such as free hemoglobin, haptoglobin, bilirubin, or LDH revealed the presence of mild, moderate, or even severe hemolysis in the majority of ECMO patients examined. When the NSE values measured simultaneously were examined, a remarkable correlation emerged between rising NSE values and escalating severity of hemolysis. On average, the NSE values of patients with severe hemolysis were more than twice as high as those of patients with mild hemolysis (Figure 5). In contrast to the stratification based on brain damage, the stratification based on the degree of hemolysis demonstrated a statistically significant difference in NSE values. The following confidence intervals and effect size resulted when comparing patients with weak or severe hemolysis: 27.7 µg/L (95% CI: 26.1–28.3 µg/L) versus 67.3 µg/L (95% CI: 48.2–76.3 µg/L), with an r_rb_-value of 0.73.

**Figure 6:**
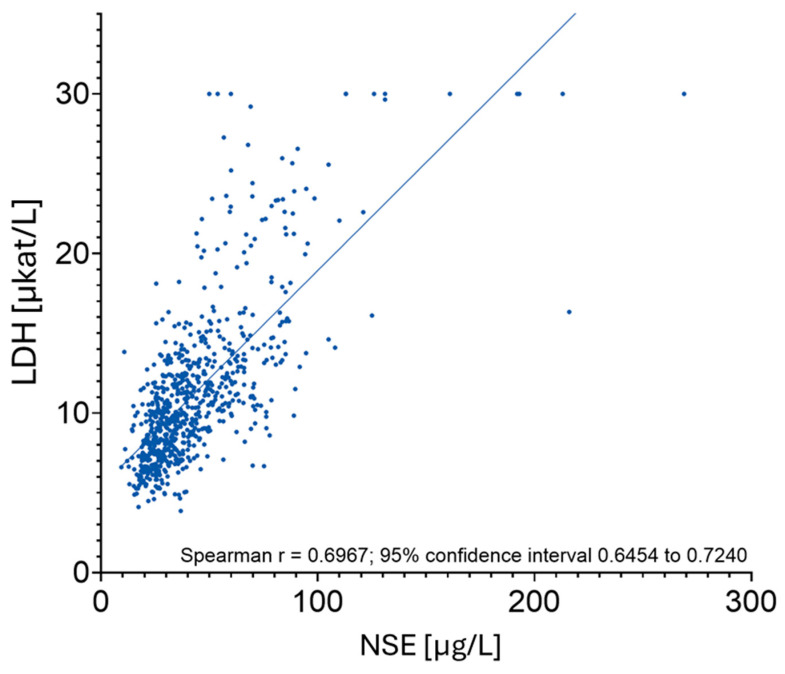
Spearman correlation of NSE and LDH values measured in parallel (*n* = 740). This analysis is based on repeated measurements of the same patients. Laboratory parameters were recorded upon admission to the ICU and then repeatedly at 24 h intervals throughout the ICU stay. Statistical analysis was performed using GraphPad Prism 10 (GraphPad Software, La Jolla, CA, USA).

A common hemolysis parameter employed in laboratory diagnostics is LDH. As a ubiquitous intracellular enzyme, LDH increases immediately in serum during the process of hemolysis, rendering it a highly sensitive marker. To assess the hypothesis of a relationship between hemolysis and NSE values, the Spearman correlation coefficient of all LDH and NSE values measured concurrently in the serum of ECMO patients was determined. A subsequent analysis of the data yielded a Spearman Rho of 0.69, which can be interpreted as a robust positive correlation between the two variables (Figure 6). The phenomenon of elevated LDH values, in conjunction with the occurrence of elevated NSE values, thus points to the presence of ECMO-related shear stress and the subsequent damage to blood cells. Consequently, in instances where elevated LDH and NSE values are detected concomitantly in ECMO patients, the diagnostic significance of NSE measurement should be regarded as constrained. Consequently, a diagnosis derived from such measurements ought to be executed with prudence.

## 3. Discussion

The utilization of extracorporeal life support therapies has exhibited a consistent upward trend in recent years [16]. Advancements in technology have not only enhanced user-friendliness but also contributed to an expansion of the safety profile, as evidenced by its extensive utilization even among patients with severe (concomitant) diseases [16]. A critical factor for the treatment outcome is the presence of neurological damage, which exerts a decisive influence on the cognitive abilities and quality of life of ECMO survivors [17,18,19,20]. The etiology of neurological impairment in patients undergoing ECMO is multifactorial, involving hypoxemia and hypo-perfusion preceding ECMO intervention. Therapy-related disturbances in coagulation and flow processes can further exacerbate this condition [17,18,19,20]. Neuro-prognosis is therefore of particular importance. The identification of brain injury in patients undergoing ECMO therapy is imperative for informed decision-making processes concerning therapy planning and communication with the patient’s family. Nevertheless, the neurological prognosis in ECMO patients poses significant challenges. A guideline-compliant clinical examination, encompassing neurophysiology and diagnostic imaging, can only be performed to a limited extent in sedated/comatose individuals and critically ill people for whom transport is risky. This underscores the role of biomarkers, which may represent a straightforward approach to neuro-prognostics.

NSE is a well-established biomarker for evaluating brain injury [3,4]. However, the NSE levels measured in patients’ blood are subject to influencing factors that affect the measured values independently of the presence or extent of neuronal damage. It has been established that extracorporeal circulation can result in hemolysis, which is concomitant with the release of NSE from blood cells [3]. Despite its immediate clinical relevance, however, there is a paucity of information in the scientific literature on the course of NSE blood levels in ECMO patients and their correlation with hemolysis parameters measured in parallel. In this retrospective study, the NSE levels of ECMO patients with and without diagnosed brain damage were compared to assess the prognostic value of NSE in the context of extracorporeal life support systems. Furthermore, the correlation between the NSE levels measured and the laboratory parameters of hemolysis was examined.

A salient finding from our analysis of repeated NSE measurements during ECMO therapy is the discovery of considerable intrapersonal variability in the measured values. This finding suggests that isolated NSE determinations may possess limited validity and interpretability. Additionally, the findings of this study indicate that elevated NSE levels, even at notably high concentrations, do not necessarily imply the presence of neurological impairment. While the NSE levels of patients with documented GCS scores of 14–15 at the time of discontinuation of ECMO support were found to be significantly lower compared to patients with GCS scores below 9, it is noteworthy that only 5.6% of patients who exceeded the recommended cut-off value of 60 µg/L, as outlined in the ERC guideline [7], had a documented brain injury. These results align with findings from a study by Lorusso et al., underscoring the low specificity of NSE for structural neurological injuries in the context of extracorporeal circulation [21]. It is noteworthy that the majority of the data in our study was derived from patients who received veno-venous therapy. The underrepresentation of patients receiving VA/VVA therapy in the present analysis hinders the ability to draw reliable conclusions regarding this patient population. Another limitation of our study is the small number of documented cases of brain damage, which restricts its statistical power. In particular, the study is underpowered for multivariate analysis. This lack of adjustment for possible confounding factors affects the interpretability of the results. While the data suggest that hemolysis obscures the utility of NSE, the small sample size prevents us from definitively rejecting its prognostic value.

The heterogeneous neurological outcomes observed in this study, even with significantly elevated NSE levels, suggest that non-neuronal sources contribute significantly to NSE blood levels during ECMO support. The initial reports identifying a correlation between NSE measurements and hemolysis emerged from cardiac surgery studies. Research conducted by Johnsson et al. in 2000 and Ishida et al. in 2003 on patients who underwent cardiopulmonary bypass surgery (CPB) demonstrated that, despite the limited sample sizes, a perioperative increase in NSE levels seemingly reflected surgery-related hemolysis (as evidenced by intraoperative increases in free hemoglobin levels) and was not associated with neurological complications [11,13]. This finding aligns with a recent study by Motoyoshi et al., which reported no statistically significant difference in NSE levels between patients with and without post-operative stroke following CPB surgery [22]. In patients undergoing extracorporeal life support therapy, system-related shear stresses have been observed to trigger hemolysis. Despite the continuous development of centrifugal pumps and membrane oxygenators to reduce flow resistance and mechanical stress, as well as the use of biocompatible coatings, hemolysis remains commonly observed among ECMO patients. As per a review study that investigates the small number of studies available on hemolysis during ECMO support, hemolysis is characterized as a prevalent occurrence, with the severity characterized as minimal in the majority of cases. Severe hemolysis (plasma-free hemoglobin >500 mg/L) is described as a rare but not exceptional event [23]. Indeed, several studies on ECMO patients support the observation of elevated NSE levels, irrespective of the presence of neurological impairment [4,5,24]. Concurrently, studies involving patients who had been implanted with a ventricular assist device (VAD) or treated with a micro-axial pump device (Impella) report a correlation between NSE levels and laboratory parameters indicating hemolysis caused by mechanical circulatory support [3,25]. In accordance with this observation, our data reveal a positive correlation between the measured NSE levels and the degree of hemolysis. In the present cohort, patients exhibiting severe hemolysis demonstrated NSE levels that were more than twice those observed in patients with only mild hemolysis. LDH was identified as a robust parameter that correlates positively with elevated NSE levels due to hemolysis.

With regard to the prognostic usefulness of NSE during ECMO therapy, our data demonstrate that even severely elevated NSE levels necessitate cautious interpretation due to potential interference with therapy-induced hemolysis. Consequently, the collection of NSE values as a component of therapeutic decision-making should invariably be incorporated within a multimodal approach.

## 4. Materials and Methods

### 4.1. Study Design and Setting

The study was conducted as a retrospective data collection and analysis at the University Medicine Halle (UMH), Germany. The monocentric project is predicated on routine data from our clinic, which is collated in the Local Research Data Portal for Health (LRDPH) at the UMH and rendered accessible for research purposes via the local Data Integration Center (DIZ). The DIZ is a collaborative initiative between the University Hospital Halle (Saale) and the Medical Faculty of the Martin Luther-University Halle-Wittenberg. This institution integrates clinical care and research by facilitating data exchange through the implementation of innovative information technology solutions. The data transfer was executed in accordance with a request and a written recommendation by the responsible Use and Access Committee. It is imperative to note that all data were provided exclusively in anonymized form. The study has been approved by the Ethics Committee of the Medical Faculty, Martin-Luther-University Halle-Wittenberg (No. 2021-146).

### 4.2. Patient Cohort

The present study incorporated data from critically ill patients who received ECMO treatment at our clinic from January 2020 to March 2022. Patients who meet the following inclusion criteria were eligible for enrollment: (1) the subject had to be at least 18 years of age; and (2) the subject had to be undergoing treatment with VV-ECMO, VA-ECMO, or VA-ECMO with Impella. Individuals who met any of the following criteria were excluded from the study: (1) resuscitation (e.g., International Classification of Diseases [ICD] codes I46.0, U60.13, I46.1, I46.9) during hospitalization, and (2) NSE value(s) not recorded.

The primary focus of the study was on the documentation and comparison of NSE values in patients with and without intratherapeutic brain damage (i.e., intracerebral hemorrhage, ischemic stroke) or clinically elevated values of the Glasgow Coma Scale at the time of termination of ECMO therapy. Secondary outcomes encompassed the correlation of NSE values with onset of hemolysis. To this end, a score was developed to evaluate hemolysis based on serum laboratory values for free hemoglobin, haptoglobin, bilirubin, and LDH, which are among the typical hemolysis parameters. In laboratory diagnostics, there is no single “gold standard” test that definitively proves hemolysis. Instead, the diagnosis is made based on a combination of various laboratory parameters (hemolysis panel) and the resulting constellation of findings. Our score replicates the plausibility check used in clinical practice based on the pattern of findings. The score’s pattern recognition feature ensures accurate interpretation by avoiding misinterpretations caused by isolated, high individual values. Instead, the measured values are weighted based on the extent of deviation from the normal value, and then evaluated in relation to each other. Known non-hemolytic confounders, such as tissue damage or hepatic dysfunction due to trauma, active malignancies, or hepatic coma, were absent in our cohort. Therefore, such influences on the measured values could be ruled out. Given the varying sensitivity of different laboratory parameters to hemolysis, specific interference limits were defined for each test based on the reference range provided by the laboratory conducting the analysis. Minor deviations from those reference ranges were assigned a score of 1, which corresponds to the following cutoff values: free hemoglobin (8–15 µmol/L), haptoglobin (0.3–0.15 g/L), bilirubin (21–50 µmol/L), and LDH (4.2–10 µkat/L). Severe deviations from the reference range specified by the analysis laboratory were assigned a score of 2 points (i.e., measured values for free hemoglobin >15 µmol/L, for haptoglobin <0.15 g/L, for bilirubin >50 µmol/L, for LDH >10 µkat/L). The total score was derived by calculating the sum of each subscore of the laboratory values for free hemoglobin, haptoglobin, bilirubin, and LDH. Scores ranging from 0 to 1 were classified as mild hemolysis, scores ranging from 2 to 5 were classified as moderate hemolysis, and scores ranging from 6 to 8 were classified as severe hemolysis.

Additionally, a comprehensive array of data elements was collected and systematically analyzed, encompassing patient characteristics (e.g., age, gender, BMI), patient outcome, the underlying cause of the patient’s condition, the specific type of ECMO therapy administered, the cannulation procedures employed, and the duration of ECMO therapy, as well as the implementation of renal replacement therapy.

### 4.3. Blood Collection and Analysis

As part of routine clinical practice, blood samples were collected from patients upon their ICU admission and then every 24 h during their stay to determine the following laboratory parameters: NSE, free hemoglobin, haptoglobin, bilirubin, and LDH. Routine collection and documentation of hemolysis parameters ensured that a complete data set was available for the retrospective analysis.

The laboratory analyses were performed in a standardized manner by the central laboratory of the University Hospital Halle using Roche cobas^®^ analysis systems (Roche Deutschland Holding GmbH, Mannheim, Germany), as recommended by the manufacturer. Specifically, the following instruments and methods were used: (1) NSE via electrochemiluminescence immunoassay (ECLIA) using the Roche cobas e 801 module; (2) free hemoglobin via the Harboe method using the Roche cobas c 502 module; (3) haptoglobin via an immunological turbidity test using the Roche cobas c 502 module; (4) bilirubin via a colorimetric diazo method using the Roche cobas c 701 module; and (5) LDH via a UV test according to the International Federation of Clinical Chemistry and Laboratory Medicine (IFCC) using the Roche cobas c 701 module.

### 4.4. Statistical Analysis

The data analysis was conducted in accordance with a predetermined plan. The initial step entailed the calculation of descriptive statistics for all variables, both in their totality and in relation to specific outcome groups. This was followed by univariable comparisons, which employed the Mann–Whitney U test, with a significance level set at *p* < 0.05. Furthermore, the confidence interval was determined, and the effect size was calculated using rank-biserial correlation. The third step involved the investigation of correlations through the implementation of a Spearman correlation analysis. All data management, statistical analysis, numerical calculations, and figure construction were conducted using GraphPad Prism version 10.0.0 (GraphPad Software, La Jolla, CA, USA).

## 5. Conclusions

In conclusion, our findings highlight the risk of misinterpreting high NSE levels as evidence of irreversible neurological injury, leading to withdrawal of life-sustaining therapy. A considerable proportion of patients with NSE levels exceeding commonly referenced neurological thresholds, i.e., above 60 µg/L, were ultimately discharged alive and some remained neurologically intact, confirming that NSE cut-off-values validated in cardiac arrest patients cannot be extrapolated to ECMO patients. The heterogeneity of ECMO modalities, cannulation strategies, duration of support, and underlying disease severity further complicates the use of NSE in neuro-prognostication. Circuit components, flow regimes and oxygenator performance influence hemolysis rates, and may introduce additional variability into biomarker levels. These confounders must be taken into account when interpreting laboratory parameters in ECMO patients and speak against NSE as a sole determinant for critical decisions in intensive care medicine, such as withdrawal of life-sustaining therapy.

## Data Availability

The raw data supporting the conclusions of this article will be made available by the authors on request.

## Abbreviations

The following abbreviations are used in this manuscript:

ARDS	Acute respiratory distress syndrome
BMI	Body mass index
CRRT	Continuous renal replacement therapy
ECMO	Extracorporeal membrane oxygenation
GCS	Glasgow Coma Scale
ICD	International Statistical Classification of Diseases and Related Health Problems
ICU	Intensive care unit
IHD	Intermittent hemodialysis
IQR	Inter quartile range
LDH	Lactate dehydrogenase
NSE	Neuron-specific enolase
VA	Veno-arterial
VAD	Ventricular assist device
VVA	Veno-veno-arterial
VV	Veno-venous
